# Automated Discovery of Food Webs from Ecological Data Using Logic-Based Machine Learning

**DOI:** 10.1371/journal.pone.0029028

**Published:** 2011-12-29

**Authors:** David A. Bohan, Geoffrey Caron-Lormier, Stephen Muggleton, Alan Raybould, Alireza Tamaddoni-Nezhad

**Affiliations:** 1 Rothamsted Research, West Common, Harpenden, Herts, United Kingdom; 2 INRA, UMR 1210 Biologie et Gestion des Adventices, BP 86510, Dijon, France; 3 Computational Bioinformatics Laboratory, Department of Computing, Imperial College London, London, United Kingdom; 4 Syngenta, Jealott's Hill International Research Centre, Bracknell, Berks, United Kingdom; Swansea University, United Kingdom

## Abstract

Networks of trophic links (food webs) are used to describe and understand mechanistic routes for translocation of energy (biomass) between species. However, a relatively low proportion of ecosystems have been studied using food web approaches due to difficulties in making observations on large numbers of species. In this paper we demonstrate that Machine Learning of food webs, using a logic-based approach called A/ILP, can generate plausible and testable food webs from field sample data. Our example data come from a national-scale Vortis suction sampling of invertebrates from arable fields in Great Britain. We found that 45 invertebrate species or taxa, representing approximately 25% of the sample and about 74% of the invertebrate individuals included in the learning, were hypothesized to be linked. As might be expected, detritivore Collembola were consistently the most important prey. Generalist and omnivorous carabid beetles were hypothesized to be the dominant predators of the system. We were, however, surprised by the importance of carabid larvae suggested by the machine learning as predators of a wide variety of prey. High probability links were hypothesized for widespread, potentially destabilizing, intra-guild predation; predictions that could be experimentally tested. Many of the high probability links in the model have already been observed or suggested for this system, supporting our contention that A/ILP learning can produce plausible food webs from sample data, independent of our preconceptions about “who eats whom.” Well-characterised links in the literature correspond with links ascribed with high probability through A/ILP. We believe that this very general Machine Learning approach has great power and could be used to extend and test our current theories of agricultural ecosystem dynamics and function. In particular, we believe it could be used to support the development of a wider theory of ecosystem responses to environmental change.

## Introduction

Ecosystems are structured by flows of energy (biomass) between primary producer plants (autotrophs) and consumers (heterotrophs), such as invertebrates, mammals and birds [1], [2]. Networks of trophic links (food webs), which are the primary routes for translocation of energy between species, are therefore very important for explaining ecosystem structure and dynamics and may lead to general theories about responses of ecosystems to environmental change [3]–[5]. Few ecosystems have been described and detailed using food webs because establishing predation relationships between the many hundreds of species in an ecosystem is resource intensive, requiring considerable investment in field observation and laboratory experimentation. Increasing the efficiency of searches for trophic links by filtering out unlikely interactions is also often not possible because of uncertainty about basic background knowledge of the network, such as whether any two species are likely even to come into contact and interact. In addition, it may require considerable analysis and interpretation to translate from the ecological ‘language’ of sample data (count, abundance, density, etc.) to the network language of links within a trophic network. Consequently, of the few ecosystems that have been studied using trophic network approaches, component communities of ecosystems that provide known, valuable ecosystem services or that are under threat have most often been evaluated [6].

Machine Learning has the potential to address many challenging problems in the ecological sciences [7]. In this paper we demonstrate that a variant of Machine Learning, Abductive ILP (A/ILP), can be used to automate the discovery of trophic links from already available sample data. The sample data we use for training come from the arable farmland ecosystem where disturbance and farm management has led to great increases in crop productivity, but often at cost to biodiversity. Here, there is concern that the extent of biodiversity loss that has occurred [8] might prevent ecosystem services, such as pollination and biological control, from being delivered [9], [10]. In this system, management disturbs trophic links, leading to the observed changes in diversity of the ecosystem [4], [11]. The hope is that by evaluating trophic links, and their sensitivity to management, trophic networks might provide a mechanism for predicting ecosystem change [12].

The data-set was sampled from 266 fields in the Farm Scale Evaluations (FSE) of genetically modified, herbicide-tolerant (GMHT) crops. This national-scale experiment evaluated the change in weed plants and invertebrates between the current, conventional herbicide management of spring-sown Maize, Beet and Oilseed Rape and winter-sown Oilseed Rape, and the herbicide management of GMHT varieties of the same crops using a split-field design [13]. We use data from the Vortis suction sampling protocol for epigeal invertebrates [14], [15]. The Vortis samples include a wide variety of generalist predators, such as carabids and spiders that are considered to be important natural enemies of pests [16], which have diverse trophic interactions that are difficult to study [17], certainly in comparison to parasitoids and pollinators. From changes in abundance of the epigeal invertebrates we calculate a geometric treatment effect ratio (for the GMHT treatment count divided by the conventional treatment count for each species or taxon in the data-set), *R*, which we treat as our primary observational data for A/ILP learning.

Firbank et al. [18] previously found no effect of the GMHT crops themselves on weeds and invertebrates, and the data can be treated as the comparison between two herbicide treatments [19]. Surface dwelling invertebrates are typically not directly affected by herbicides, but are affected through the indirect effects of changes in resources mediated by the loss of weed plant food and shelter [14], [20]. To construct a hypothetical trophic network for the Vortis data using A/ILP, we develop a simple conceptual model for the specific example of the change in epigeal invertebrates between the conventional and GMHT half-fields; appropriate conceptual models would need to be developed to allow the application of A/ILP on other data-sets. We presume that the difference between the halves of each split field is due to management-induced mortality of weed plants perturbing the food supply or refugia of epigeal invertebrate herbivores and detritivores. These animals then either die, *in situ*, or relocate to other host weed plants, possibly in the contrasted treatment. The predators of these herbivores and detritivores may also relocate, possibly in response to their prey items. Consequently, one could induce that species redistribution across the two treatments, following the perturbation of the system by management, would happen such that their treatment *R*-ratios were directly correlated.

Alone, correlated *R*-values might lead to fairly poor discrimination of trophic links because there are many possible interactions, such as competition or reproduction, which might lead to correlation. A/ILP methods could be used to explore and hypothesize the effects of these different interaction processes on network structure. Here, however, we identify candidate species pairs with correlated *R*-values that are trophically linked from those that are not, using ‘background knowledge’. Trophically linked species should share a number of properties that non-linked species should not. These properties would include an expectation that at least one of the species pair could be considered a predator; herbivores or species with inappropriate mouthparts cannot be predators. We also expect putative predators to be larger than their prey [21]. Finally, it is expected that the prey and predator co-occur within the sample, being found within the individual Vortis samples that make up the half-field data-sets. This background knowledge acts as conditions on the pairwise species data selecting for combinations that we predict would be trophically linked. Importantly, these trophic hypotheses arise from the data and background knowledge, and independent of preconceptions, such as ‘species A must eat species B’.

To derive the trophic hypotheses, we use Inductive Logic Programming (ILP) [22], a form of Machine Learning that uses a logical representation to describe hypotheses derived from encoded observation and background knowledge. Problems of network construction similar to learning food webs have been tackled in other complex systems, such as gene and metabolic networks, using an Abductive variant of ILP [23]. Here, we demonstrate that A/ILP can generate plausible and testable hypotheses for ‘who eats whom’ from ecological data. In this approach the abductive predicate ‘eats’ is entirely undefined before the learning begins. This contrasts with previous applications of A/ILP where partial, non-empty, definitions exist and the gaps are filled by abduced hypotheses. We also demonstrate a new approach for estimating probabilities for hypothetical ‘eats’ relations based on their frequency of occurrence when random permutations of our ecological ‘training’ data (and hence different seeds for defining the hypothesis space) are considered.

Our goal for this methodology is to develop and test generic theory for the predictability of ecosystem change following perturbation. Models of single species undergoing perturbation have some value, but tend to be limited in their generality because a single species model does not teach us much about what the models for other species, or groups of species, should look like [4], [5]. We would like to make system-wide predictions, across many species, for ecosystem structure and functioning based on generic network theory. In this paper we develop the logic and hypothesize a heterotrophic network from *R*-values taken from Vortis suction sample data, and provide evidence in support of the veracity of the hypothesized links from the literature, where this is possible. We then discuss the value of the method for this example and its application in Ecology.

## Methods

### Abductive reasoning and A/ILP

The main role of abductive reasoning in machine learning of scientific theories is to provide hypothetical explanations of empirical observations [24]. Then, based on these explanations, we try to inject back into the scientific theory new information that helps complete the theory. This process of generating abductive explanations and updating theory can be repeated several times as new observational data become available. In many implementations of abductive reasoning, such as that of Progol 5.0 used in this paper [25], the approach taken is to choose the explanation that ‘best’ generalizes under some form of inductive reasoning. This link to induction then strengthens the role of abduction to machine learning and the development of scientific theories. We refer to this approach as Abductive ILP (A/ILP). Technically we refer to induction as a process of taking a set of examples encoded as logical sentences that are free of variables and replacing them with more general hypotheses expressed as logically encoded sentences that contain universally quantified variables. By contrast, in abduction the hypotheses are also free of variables, and thus cannot be viewed as general rules since they do not contain universally quantified variables. A/ILP technology supports both abductive and inductive generalisation. In the present application we use an A/ILP system, Progol5.0, in abductive mode to construct food webs. Progol 5.0, is freely available for academic purposes.

Given a theory, *T*, that describes our incomplete knowledge of the scientific domain and a set of observations, *O*, we can use abduction to extend the current theory according to the new information contained in *O*. The abduction generates hypotheses that entail a set of experimental observations subject to the extended theory being self-consistent. Here entailment and consistency refer to the corresponding notions in formal logic. Abduction is typically applied to problems that can be separated into two disjoint sets of predicates: the *observable* predicates and the *abducible* predicates. In practice, observable predicates describe the empirical observations of the domain that we are trying to model. The abducible predicates describe underlying relations in our model that are not observable directly but can, through the theory *T*, bring about observable information. Hence, the hypothesis language (i.e. abducibles) can be disjoint from the observation language. We may also have background predicates (prior knowledge), which are auxiliary relations that help us link observable and abducible information.

### FSE data

The FSEs were conducted across Great Britain (GB) in 266 arable fields [15], [26]. Site selection was designed to provide fields that were representative of the spectrum of current arable cropping in GB, in terms of environmental and agronomic variables [15], [26]. A total of 68, 67 and 66 fields of spring-sown maize, oilseed rape and beet, respectively, and 65 fields of winter-sown oilseed rape were selected. Each field was split in half, and one half was sown with a conventional crop variety and the other with the test GMHT variety [27]. Invertebrate and weed sampling was conducted at fixed sampling points along some or all of 12 transects in each half-field, each 32 m long and running perpendicularly from the field edge into the field [13]–[15], [28]. Details of the Vortis protocol, freely available from the Royal Society Publishing website [14], [15], are only briefly described here.

For the invertebrates we use year total, species and taxon counts of invertebrates sampled using a Vortis suction sampler from the surface of the weeds and soil [14], [15]. In each half-field, five 10 second suction samples, spaced 1 m apart, were taken at 2 and 32 m along three transects into the crop. For the spring-sown crops, samples were taken in June and August, while samples from winter oilseed rape were taken in September/October, and May/June. Some invertebrates could not be identified to species, and these were grouped into higher order taxa. Identification was done to the taxonomic levels specified in Table 1 of Roy et al. [29]. Counts of the invertebrate species or taxa were summed across the sampling points in each half-field and then across the sampling dates to achieve a year total count for each species or taxon in each half-field.

We note that population dynamic theory and empirical evidence [30] suggest that time delays, or lag, in redistribution could significantly disrupt our expected model of positively correlated *R*-values presented in the Introduction. In the FSE, the sampling of invertebrates was done, mindful of such potential disruption, by taking samples one week or more after the treatment-level conventional and GMHT herbicide managements were done [13], [15].

The counts from each conventional and GMHT half-field pair were converted to a geometric treatment ratio, as used in Haughton et al. [14]. Counts were log-transformed, using formula L_ij_ = log_10_(C_ij_+1), where C_ij_ is count for a species or taxon in treatment i at site j. Sites where (C_1j_+C_2j_)≤1 were removed from the learning data-set (as in [14]). The treatment ratio, *R*, was then calculated as R = 10^d^ where d = (L_2j_−L_1j_). Following the rationale in Squire et al. [31], important differences in count between the two treatments were considered to be greater than 50%. Thus, treatment ratio values of *R*<0.67 and *R*>1.5 were regarded as important differences in count with direction of *down* (*decreased*) and *up* (*increased*) in the GMHT treatment, respectively. This information on *up* and *down* abundances is regarded as our primary *observational* data (*O*) for the learning.

### Background or Prior knowledge

#### Trophic behaviour

Some 181 species or taxa, totalling 193,558 individuals, from the Vortis sampling were included for A/ILP learning. These species and taxa were allocated either to consumer or non-consumer groupings, based upon the work of Hawes et al. [32], prior knowledge and expert opinion of Agricultural Entomologists and Ecologists.

#### Body size

Each species or taxon in the data-set was allocated to a body size category on a scale from small (size class 1) to large individuals (size class 4) [4]. This categorization was based either upon the length of the species found in the literature or expert opinion of length relative to those already categorized. It should be noted that this estimate of body size, based upon length, does not take account of body plan and so may be a poor surrogate for body mass.

#### Co-occurrence

Co-occurrence scores were computed for each species or taxon combination from the Vortis data-set. The co-occurrence scores were achieved at each of the sampling points, at 2 m and 32 m, on the three transects in each half-field. Any two species were scored as co-occurring at a sample point where the count for both species was 1 or greater.

### Machine learning of trophic relations from FSE data

We believe that ecological data in this study fulfil the conditions for the use of A/ILP: firstly, the given background knowledge is incomplete; and secondly, the problem requires learning in the circumstance in which the hypothesis language is disjoint from the observation language. In our problem, the set of FSE *observable* data can be compiled and represented by predicate *abundance(X, S, up)* or *abundance(X, S, down)*, expressing the fact that the relative abundance of species *X* at site *S* is *up* or *down*, in the GMHT treatment. The knowledge gap that we initially aim to fill is a trophic relationship between species. Thus, we declare abducible predicate *eats(X, Y)* capturing the hypothesis that species *X* eats species *Y*. In order to use abduction, we also need to provide the rules that describe the observable predicate in terms of the abducible predicate. An example of such a rule is shown below.

*abundance(X, S, up)* ***if***
*predator(X) and*
*co_occurs(S, X, Y) and*
*bigger_than(X, Y) and*
*abundance(Y, S, up) and*
*eats(X, Y).*

Similarly, a rule for *abundance(X, S, down)* can be defined. This rule expresses the inference that following a management-driven perturbation in the ecosystem, the changed abundance of species *X* at site *S* can be explained by the fact that *X* eats species *Y* which is further down in the food chain and the change in the abundance of species *Y*. It also includes additional conditions to constrain the search for abducible predicate *eats(X, Y)*. These constraints are that *X* should be a predator, *X* and *Y* should co-occur and that *X* should be bigger than *Y*. Predicates *predator(X)* and *bigger_than(X, Y)* are provided as part of the background knowledge and *co_occurs(S, X, Y)* is compiled directly from FSE data. This model describes at an appropriately high level the possible transitive effect of management leading to increased or decreased abundance of species.

Given the A/ILP model described in this section and the observed FSE data, Progol 5.0 generates a set of abductive hypotheses in the form of *eats* relations between species. To achieve probability estimates for these hypothetical *eats* relations, we use a technique that is based on direct sampling from the hypothesis space. In some ILP systems, including Progol 5.0, training data also act as seeds to define the hypothesis space. Hence, different permutations of the training examples define different parts of the hypothesis space. We use this property to sample from the hypothesis space by random permutations of the training data. The probability of any given hypothetical *eats* relation can be estimated from its frequency of occurrence across random permutations of the training data (and hence different seeds for defining the hypothesis space).

To formally evaluate the predictive power of the hypothetical trophic links, we use a ‘leave-one-out’ cross-validation test on the observed data for species in the network. The abundance of each predator at each site is left out of the training in turn and we try to predict whether the abundance of the excluded species is *up* or *down*, given the trophic network generated from the remainder of the data. We report the average *predictive* accuracy, defined as the proportion of correctly predicted left-out test examples. We also report standard errors associated with predictive accuracies. To ascertain whether the inclusion of probability estimates for each ‘eats’ relation would have value, we use relative frequencies in the same way probabilities are used in probabilistic ILP [33]. We calculate the relative frequencies for hypotheses that imply the abundance of a test example is *up* and if this is higher than the relative frequencies which imply that the test example is *down* then we predict that the abundance of the test example is *up*, otherwise it is *down*.

### Corroboration of the hypothesized food web

The veracity of the hypothesized network was examined using a literature search, the result of which is presented as a figure in the text and a reference list in the supplementary materials (File S1). The quality of the information cited varies, however. In some cases a reference describes direct tests of the hypothesized species interaction using either gut dissections or molecular diagnostics on gut contents. This provides the hardest evidence. Other papers relate to observational studies where two species have been observed interacting and feeding has either been observed or presumed. For the main body of the papers, the evidence is anecdotal. Authors have assumed the link exists and analysed field data based on this assumption. This category of expert opinion provides the weakest evidence and is provided to show that these links are accepted as possibilities. Coccinelids (ladybirds or ladybugs) are extremely polyphagous consumers [34]. Expert opinion from a specialist was sought to determine whether a potential prey item might therefore fit the bill of fare. For the larger carabids, such as the *Trechus* and *Bembidion* species, we have presumed that reference to a prey item of any one species within either of these genera may also be taken as evidence of predation for all species within the genera. This paper therefore presents corroborative evidence for our hypothetical links being realistic rather than being strict tests of those hypotheses.

## Results

**Figure 1:**
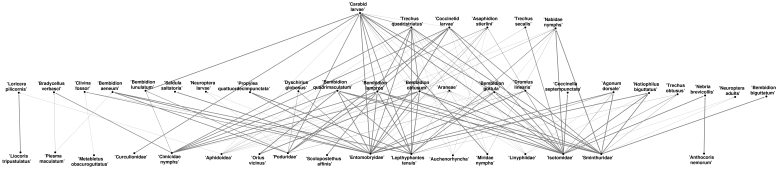
Trophic network hypothesized by A/ILP from Vortis sampled invertebrates in the FSE data-set. Each link between a species or taxon represents a learnt ‘eats’ relation that could be tested either against the literature or by experimentation. The thickness of the link indicates the estimated probability of occurrence, based on the relative frequency from 10 random permutations of the FSE training data.

**Figure 2:**
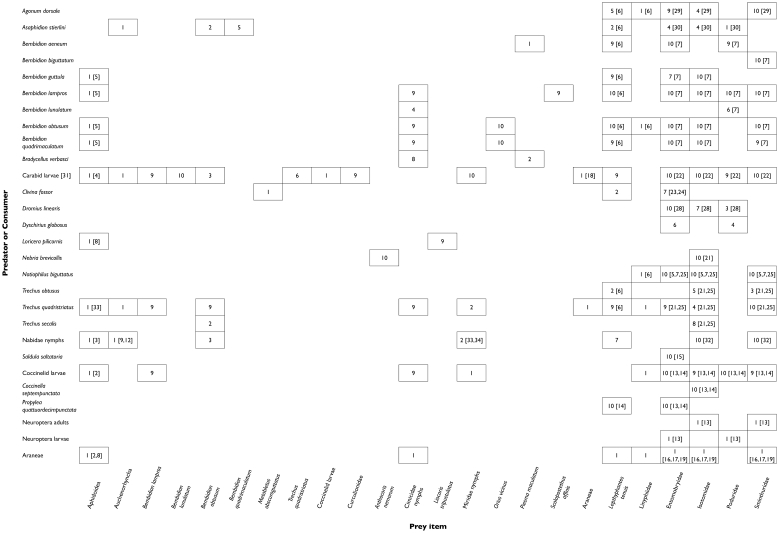
Representation of the links hypothesized for each prey item and consumer species or taxon combination in **Figure 1**. Each pairwise expectation has a permuted probability (relative frequency), presented as link thickness in Figure 1, and reference numbers, in square brackets, for references listed in the supplementary materials [File S1].

Given the observed data and the model described in the previous sections, Progol 5.0 generates a set of hypotheses in the form of ‘eats’ relations between species. This set of hypotheses can be visualised as a network of trophic links (food web) shown in Figure 1. In this network a relation *eats(X, Y)* is represented by a trophic link from species *Y* to *X*. The thickness of trophic links represent the probabilities associated with each hypothetical ‘eats’ relation estimated from the frequency of their occurrence in 10 random permutations of the training data (Figure 1; Figure 2).

The predictive accuracy of probabilistic networks, generated from 10 random permutations, was found to be 73.67%±2.55. This was significantly greater than for non-probabilistic networks (65.33%±2.75) or those constructed from 10 random permutations but without the inclusion of probabilities (64.67%±2.76). In all cases the predictive accuracies were significantly higher than the default accuracy of the majority class (i.e. 51.7%).

### Species counts

45 species or taxa were hypothesized to be important within the Vortis sampled trophic network, representing some 144,061 individuals across the conventional and GMHT half-fields (Figure 1). This number represented approximately 25% of the species or taxa and about 74% of the invertebrate individuals included in the learning.

The full details of the abundance and diversity of the Vortis sampled invertebrates were presented in Haughton et al. [14] and Bohan et al. [15].

### Network structure

Large carabids, including *Bembidion* sp., *Trechus* sp. and *Nebria brevicollis*, were found to be important components of the network, being strongly associated with entomobryid, isotomid, podurid and sminthurid Collembola prey items (Figure 1, Figure 2). Coccinelid larvae were also hypothesized to prey upon these collembolans, and with the *Bembidion* sp. and *Trechus quadristriatus* on nymphal stages of the Cimicidae. The network structure also suggests that certain predatory invertebrates, such as *Bembidion lampros*, heteropteran Cimicidae larvae and the spider *Lepthyphantes tenuis*, may also serve as intra-guild predation (IGP) prey items for other *Bembidion* sp., coccinelid larvae and *T. quadristriatus*. Possibly the most important consumers within the hypothesized network were the carabid larvae which are expected to have strong relationships with a number of prey item species and taxa. The detritivore Collembola, represented as the broad taxonomic groupings of the Entomobryidae, Isotomidae, Poduridae and Smithuridae, were consistently hypothesized to be important prey resources for a wide variety of predatory species and taxa, and particularly the generalist and omnivorous carabids. Relationships between aphid prey, which represent major prey resources, and potential aphid predators were present but unexpectedly weak.

We found evidence in the literature to support many of the hypothesized trophic relationships present within the Vortis network (Figure 2).

## Discussion

We find that machine learning, using A/ILP, produced a convincing food web from available Vortis sample ecological data. Many of the learnt trophic links are supported either by information gathered from the literature or the expert knowledge of Agricultural Ecologists. This A/ILP food web was built using logical statements for interactions between species that are expected to be trophic, encoded in Progol 5.0, which can readily be interpreted by Ecologists. This means that the logic framework for learning trophic links, or ‘eats’ relations, can be openly discussed, *a priori*, and the hypothesized links are not an abstract, statistical product of the data. Two aspects of the use of A/ILP in this paper are particularly novel. Firstly, the abductive predicate ‘eats’ is entirely undefined before the start of the learning. This contrasts with previous applications of A/ILP [23] in which knowledge gaps exist in a partial, non-empty definition and are filled by abduced hypotheses. This setting is close to the classic hard problem of predicate invention within Inductive Logic Programming. The second novel aspect of the approach relates to the assignment of probabilities to hypothetical ‘eats’ relations based on their frequency of occurrence when randomly sampling the hypothesis space. The resulting probabilistic network is a compact summary of the hypothesis space with a posterior distribution that could be viewed as a Bayes predictor, and is expected to have lower error [35]. The results of cross-validation tests suggest that the trophic networks with probabilities have significantly higher predictive accuracies compared to the networks without probabilities. Using probabilities helps to separate those trophic links with low probabilities, which represent unstable artefacts, possibly of ordering in the data-set, from those with high probabilities that can be viewed as stable and reliable hypotheses.

The results we present are individual, hypothetical ‘eats’ relations assembled into a candidate heterotrophic, arable food web that is relevant to the GB national scale. This web is for the epigeal [14] component of the invertebrates present within the arable system and it allows us to reject, or not, each hypothesized trophic link. The detritivore Collembola are hypothesized to be the major prey items within the putative network, as expected from direct observation [36]–[38]. The learnt food web suggests that large generalist or omnivorous carabid beetles were the predominant predators within the epigeal component [39], [40]; an expectation also supported by their relatively high abundance in the Vortis sample [14], [15]. Members of the *Bembidion* and *Trechus* genera and *N. brevicollis* were hypothesized to prey upon a variety of species and taxa, including one another.

Discovered trophic links might be tested formally using molecular diagnostics and more traditional gut dissections and observational studies. Beyond an acceptable period of formal testing to show that the automated discovery methods produce valuable information for different situations and species combinations, repeated testing of whole networks would miss the value of this approach. Automated discovery will have most value when it is used to generate networks without the burden of observation that is currently required for food web construction. After the method has ‘proved its mettle’, however, such network learning and generation will still require some level of testing and verification. This should probably be limited to testing links that were not expected rather than extensive retesting of well-established trophic interactions.

The physical structure of the food web is in part a consequence of the partial background information. For the ‘eats’ relations, we stated an expectation that invertebrate predators should be larger than their prey [21]. In effect, ‘big things eat small things’ [4]. Given that we assigned each species or taxon to a 4-level body size class, this means that the web is limited to four trophic levels. Consequently, relatively big organisms, such as carabid larvae, have a large pool of potential prey to draw upon. Despite this, we were surprised at the number, range and strength of the links predicted between carabid larvae predators and smaller prey items. Indeed this food web would suggest that carabid larvae are an extremely important predator group amongst epigeal invertebrates. While carabid larvae are known as voracious, generalist predators [41], [42], difficulties in sampling and an often subterranean habit has limited our knowledge of their predatory role within arable farmland. In a recent paper, however, Eitzinger and Traugott [42] have demonstrated that larvae of *N. brevicollis* have a wide prey range, including Collembola and linyphiid spiders. This A/ILP learning suggests that carabid larvae trophic behaviour is evident in Vortis sample data, even though this method does not sample below ground, and generates a series of future hypotheses for trophic interactions between carabid larvae and possible prey items that could test the importance of carabid larvae as predators within the arable system.

A noticeable feature of the hypothesized food web is the widespread presence of trophic links within the guild of predators. For example, the A/ILP suggests that there are links between carabid larvae and adults (*Bembidion* sp.), the bembidions and spiders (*e.g. L. tenuis*), Cimicidae nymphs and *Orius vicinus*, *Trechus* sp. and bembidions, cimicid nymphs, Miridae nymphs and spiders. Intra-guild predation can modify the structuring and dynamics of a trophic network [43], in addition to reducing the efficacy of prey control [44]. IGP may be widespread within arthropod predator communities [44]–[47]. However, except for a few cases, and particularly for IGP involving the heteropteran bugs, there is little evidence from the literature to support these particular links. It would be an extremely valuable exercise to determining observationally whether the hypothesized IGP links have any value, and might adversely affect pest control functions provided by invertebrate predators in farmland.

The trophic network does highlight a problem with our expectation that big things eat small things. Spiders appear in the network only as prey items, except for a low probability entry as predators of aphids, Cimicidae nymphs and the collembolans. Spiders sampled by the Vortis, such as *L. tenuis*, have low body size. However, spiders are obligate predators. The positioning of spiders in the network might reflect the treatment of the Vortis data-set in isolation. Spiders might be linked, as predators, to other species not sampled by the Vortis suction sampling protocol. By using silken webs, some spiders may also capture prey much larger than themselves. While there is evidence that spiders do form the prey of larger carabids (see supplementary materials File S1), which would support the food web as presented, further thought is necessary for how to incorporate groups that might not obey our simple background information expectations. It would be necessary to test whether the dimensions of a spider web might be a more valid measure of spider trophic size than body length.

The size condition used in the model leads to uni-directional trophic links. One species assumes the trophic role of the consumer and one the prey item. The possibility that the interaction is more symmetrical, with either species being able to consume the other depending on a particular set of circumstances, is excluded. Potentially, this constraint might lead to the generation of unrealistic food webs, particularly for groups like the spiders. Body size determines the likely trophic role in any interaction and for smaller organisms this might lead to an increased rate of false negatives for trophic links.

The methodology for learning ‘eats’ relations relies heavily on correlation between *R*-values. Correlated *R*-values alone would, however, lead to fairly poor discrimination of trophism between any pair of species in the data-set. Such correlations could come about in species that share common food resources. They might also arise simply through chance. It is the background information, such as expectations of body size relationships and whether a species might be a predator or not, that allows us to propose a trophic model and learn who eats whom from this potentially confounded data. However, this does not explain why we have not learnt trophic links between species that we expect to eat one another from field observations. The Aphidoidea are prey items in a number of hypothesized trophic links, but all are ascribed with low probability. Field experience would suggest that aphids are important food resources for a number of predator groups [39], [48], [49], including *Agonum dorsale*. This lack of strong eats relations may be due to a number of reasons that change the variation and correlation-values of *R* across sites. One or both of the species or taxa being considered may not depend on the herbicide management being used to perturb the ecosystem. Those that largely reside or feed on the herbicide-unaffected crop plants might be insensitive to perturbations caused by herbicide management, such as some species of aphid pests of the crop. Certain species may also be affected by insecticide sprays applied to control pest numbers; disturbances that are not taken into account here. In addition, the Vortis protocol itself is selective and does not appropriately sample some species within the network [14]. By example, we found extremely low numbers of *A. dorsale* in the Vortis suggesting that this might not be an appropriate sampling method for this species.

The hypothesized Vortis network contains a high proportion of generalist species with a relatively high density of links, and IGP, compared to specialist links involving isolated pairs of species. This is largely an artefact of the probabilistic nature of the network, which is built by superimposing many individual food webs estimated from permutations of the data. Within the non-probabilistic networks, from which the final probabilistic model was constructed, we find isolated interactions between species, much like those found in traditional host-parasitoid food webs [12], [50]. High link-density and IGP might also result from the way that we have treated the Vortis data in developing this learning methodology. To keep the method development manageable, we examined the Vortis data in isolation, excluding other predators, including mammals and birds, which might impact on the food web structure by, potentially, reducing link density and IGP. The statistics for predictive accuracy, however, would not indicate that such effects are large. Specialist interactions are also highly sensitive to the exclusion of either predator or prey species, as might happen to those species not sampled, or not sampled well, using Vortis. We have also treated crop and other factors, such as location and management, as random sources of variation that we assumed would not affect the hypothesized links and structure of the network. While our experience of the FSE data would tend to support this assumption for many such random sources of variation (see for example [20], [51], [52], we would not expect this to hold for crop type as Vortis species composition is known to vary systematically between crops [53]. It may be that the structure we have learnt here therefore reflects those links that appear in all cropping situations; those that tend to be generalist.

In the future we will examine the sensitivity and generality of logic-based machine learning of food webs, across cropping and management situations. One goal is to examine whether these methods are general and can be directly applied to other ecological protocols, initially using data from the FSE. Tests of generality might be to examine whether, for species-pairs sampled in both the Vortis and a comparison protocol, such as pitfall trap data, Vortis eats predicates also apply with high predictive power to the other protocol. We will also examine the sensitivity of the ‘eats’ relations and the hypothesized network to changes in the values of *R* that are defined as being important. Are there critical values of ecological change beyond which the network becomes saturated or no links are apparent at all? Is the network sensitive to the sample size or population dynamic time lags? For the FSE data we can juggle with within-field and between-field data and so attempt to answer questions about appropriate sampling designs: for example, how many within-field sample points and field sites are necessary for constructing food webs? In the introduction we introduce a model that links the observed value of *R* to trophism. For other data-sets, it might not be possible to calculate values comparable to *R*. We need to know what happens if we change our descriptive model and use another metric of ecological change than *R*. We believe that this process of testing and analysis of the method will allow us to learn food webs across different protocols and potentially build a robust, ecosystem-wide food network for the UK arable agricultural ecosystem.

The value of ecological function and the theories of functional ecology for predicting system change, is currently a topic of great debate amongst Ecologists [54]. Machine learning approaches might be used to provide a test at the largest scale, greatly extending fundamental ecological theory. Using the ecosystem-wide description of the arable food web, it might be possible to ask which of species- or functionally-based descriptions yield food webs that have greater parsimony and might, therefore, be more robust predictors of the effects of environmental change on agroecosystem diversity and productivity.

## Supporting Information

File S1 List of references noted as reference numbers in Figure 2.
(DOC)
