# Supplementary material for: Automated Discovery of Food Webs from Ecological Data Using Logic-Based Machine Learning
Source: PLoS One. 2011 Dec 29;6(12):e29028. doi: 10.1371/journal.pone.0029028 (PMC3248413; doi:10.1371/journal.pone.0029028)
Supplement: File S1 — List of references noted as reference numbers in Figure 2. (DOC) [file pone.0029028.s001.doc]

**Supplementary materials**

1. Kuusk A-K, Cassel-Lundhagen A, Kvarnheden A, Ekbom B (2008) Tracking aphid predation by lycosid spiders in spring-sown cereals using PCR-based gut-content analysis. Bas Appl Ecol 9: 718-725.
2. Carter MC, Dixon FG (1982) Habitat Quality and the Foraging Behaviour of Coccinellid Larvae. J Anim Ecol 51: 865-878.
3. Pons X, Nunez E, Lumbierres B, Albajes R (2005) Epigeal aphidophagous predators and the role of alfalfa as a reservoir of aphid predators for arable crops. Eur J Entomol 102: 519-525.
4. Holland JM, Winder L, Woolley C, Alexander CJ, Perry JN (2004) The spatial dynamics of crop and ground active predatory arthropods and their aphid prey in winter wheat. Bull Ent Res 94: 419-431.
5. von Berg K (2007) The role of detrital subsidies for biological control by generalist predators evaluated by molecular gut content analysis. Dissertation von Dipl.-Biol. aus Technischen Universitat Darmstadt.
6. Dinter A (1998) Intraguild predation between erigonid spiders, lacewing larvae and carabids. J Appl Entomol 122: 163-167.
7. Sunderland KD (1975) The Diet of some Predatory Arthropods in Cereal Crops. J Appl Ecol 12: 507-515.
8. Sunderland KD, Crook NE, Stacey DL, Fuller BJ (1987) A Study of Feeding by Polyphagous Predators on Cereal Aphids Using Elisa and Gut Dissection. J Appl Ecol 24: 907-933.
9. Weiser Erlandson LA, Obrycki JJ (2010) Predation of Immature and Adult Empoasca fabae (Harris) (Hemiptera: Cicadellidae) by Three Species of Predatory Insects. J Kansas Entomol Soc 83: 1-6.
10. Martinez DG, Pienkowski RL (1982) Laboratory studies on insect predators of potato leafhopper eggs, nymphs and adults. Environ Entomol 11: 361-362.
11. Rothschild GHL (1966) A Study of a Natural Population of Conomelus anceps (Germar) (Homoptera: Delphacidae) Including Observations on Predation Using the Precipitin Test. J Anim Ecol 35: 413-434.
12. Waloff N (1980) Studies on grassland leafhoppers (Auchenorrhyncha, Homoptera) and their natural enemies. Adv Ecol Res 11: 81-215.
13. Turner BD (1984) Predation pressure on the arboreal epiphytic herbivores of larch trees in southern England. Ecol Entomol 9: 91-100.
14. Weber DC, Lundgren JG (2009) Assessing the trophic ecology of the Coccinellidae: Their roles as predators and as prey. Biol Control 51: 199-214.
15. <http://www.commanster.eu/commanster/Insects/Bugs/SpBugs/Saldula.saltatoria.html>
16. Lawrence KL, Wise DH (2000) Spider predation on forest-floor Collembola and evidence for indirect effects on decomposition. Pedobiologia 44: 33-39.
17. Agustí N, Shayler SP, Harwood JD, Vaughan IP, Sunderland KD et al. (2003) Collembola as alternative prey sustaining spiders in arable ecosystems: prey detection within predators using molecular markers. Mol Ecol 12: 3467-3475.
18. Davey JS, Vaughan IP, King RA, Bell JR, Bohan DA, Bruford MW, Holland JM, Symondson WOC (unpublished data).
19. Chapman EG, Romero SA, Harwood SA (2010) Maximizing collection and minimizing risk: does vacuum suction sampling increase the likelihood for misinterpretation of food web connections? Mol Ecol Res 10: 1023-1033.
20. T. Bilde, J. A. Axelsen, S. Toft. (2000) The value of Collembola from agricultural soils as food for a generalist predator. Journal of Applied Ecology, 37, 672–683.
21. Warner DJ, Allen-Williams LJ, Warrington S, Ferguson AW, Williams IH (2003) Mapping, characterisation, and comparison of the spatio-temporal distributions of cabbage stem flea beetle (Psylliodes chrysocephala), carabids, and Collembola in a crop of winter oilseed rape (Brassica napus). Entomol Exp Appl 109: 225-234.
22. Bauer T (1982) Prey-capture in a ground-beetle larva. Anim Behav 30: 203-208.
23. Sunderland KD, Lovei GL, Fenlon J (1995) Diets and Reproductive Phenologies of the Introduced Ground Beetles Harpalus affinis and Clivina australasiae (Coleoptera, Carabidae) in New Zealand. Aus J Zool 43: 39-50.
24. Desender K, Pollet M (1985) Ecological data on Clivina fossor (Coleoptera, Carabidae) from a pasture ecosystem II. Reproduction, biometry, biomass, wing polymorphism and feeding ecology. Rev Ecol Biol Sol 22: 233-246.
25. Bell JR, King RA, Bohan DA, Symondson WOC (2010) Spatial co-occurrence networks predict the feeding histories of polyphagous arthropod predators at field scales. Ecography 33: 64-72.
26. Holopainen JK, Helenius J (1992) Gut Contents of Ground Beetles (Col., Carabidae), and Activity of these and other Epigeal Predators during an Outbreak of Rhopalosiphum padi (Hom., Aphididae). Acta Agr Scand B-S P 42: 57-61.
27. Sunderland KD, Vickerman GP (1980) Aphid Feeding by Some Polyphagous Predators in Relation to Aphid Density in Cereal Fields. J Appl Ecol 17: 389-396.
28. Alexander KNA. (2002) The invertebrates of living and decaying timber in Britain and Ireland. English Nature Research Reports no. 467.
29. Toft S (2005) The quality of aphids as food for generalist predators: implications for natural control of aphids. Eur J Entomol 102: 371–383.
30. Bauer T, Desender K, Morwinsky T, Betz O (1998) Eye morphology reflects habitat demands in three closely related ground beetle species (Coleoptera: Carabidae). J Zool 245: 467-472.
31. B. Eitzinger, M. Traugott. (2011) Which prey sustains cold-adapted invertebrate generalist predators in arable land? Examining prey choices by molecular gut-content analysis. Journal of Applied Ecology, 48, 591–599.
32. Pons X, Lumbierres X, Albajes R (2009) Heteropterans as aphid predators in inter-mountain alfalfa. Eur J Entomol 106: 369–378.
33. Braman K (2000) Damsel bugs (Nabidae). In: Schaefer CW, Panizzi AR, editors. Heteroptera of Economic Importance. Boca Raton: CRC Press LLC. pp. 639–656.
34. Lattin JD (1989) Bionomics of Nabidae. Ann Rev Entomol 34: 383–400.
